# Poor self-rated oral health associated with poorer general health among Indigenous Australians

**DOI:** 10.1186/s12889-021-10426-3

**Published:** 2021-03-01

**Authors:** Xiangqun Ju, Joanne Hedges, Gail Garvey, Megan Smith, Karen Canfell, Lisa Jamieson

**Affiliations:** 1 Australian Research Centre for Population Oral Health, Adelaide Dental School, The University of Adelaide, Adelaide, 5005 Australia; 2 Menzies School of Health Research, Darwin, Australia; 3 Cancer Council of NSW, Woolloomooloo, Australia; 4 School of Public Health, University of Sydney, Sydney, Australia

**Keywords:** Indigenous Australians, EuroQoL (EQ-5D-5L), Disutility score, Self-rated oral health

## Abstract

**Background:**

Oral diseases negatively impact general health, affecting physical, psychological, social and emotional wellbeing, and ability to give back to community. The relationship between poor oral health, and general health and wellbeing among Indigenous Australians has not been documented. Working in partnership with seven Indigenous communities in South Australia, this study aimed to: 1) quantify self-rated oral health and health-related quality of life and; 2) investigate associations between poor self-rated oral health and general health among Indigenous Australian adults.

**Methods:**

Data was collected from a large convenience sample of Indigenous Australians aged 18+ years from Feb 2018 to Jan 2019. General health-related quality of life, as the main outcome variable, was measured by calculating disutility scores with the five individual EQ-5D dimensions (EuroQol instrument: EQ-5D-5L), then classified as ‘no problem’ and ‘at least one problem’. Self-reported oral health, as the main explanatory, was dichotomised into ‘fair or poor’ and ‘excellent, very good or good’. Multivariable log-Poisson regression models were used to estimate associations between poor self-rated oral health and general health by calculating mean rate ratios (MRR) for disutility scores and prevalence ratios (PR) for individual dimensions, after adjusting for social-demographic characteristics and health-related behaviours.

**Results:**

Data were available for 1011 Indigenous South Australian adults. The prevalence of ‘fair or poor’ self-rated oral health was 33.5%. The mean utility score was 0.82 (95% CI: 0.81–0.83). Compared with those rating their oral health as ‘excellent or very good or good’, those who rated their oral health as ‘fair or poor’ had a mean disutility score that was 1.6 (95% CI: 1.1–2.2) times higher, and the prevalence of at least one problem ranged from 90 to 160% higher for individual EQ-5D dimensions.

**Conclusions:**

Fair or poor self-rated oral health among Indigenous persons in South Australia was associated with poor general health as measured by EQ-5D-5L disutility. The relationship was especially evident with respect to mobility, self-care and anxiety/depression. The findings emphasise the importance of oral health as predictors of general health among Indigenous Australians.

**Supplementary Information:**

The online version contains supplementary material available at 10.1186/s12889-021-10426-3.

## Background

Aboriginal and Torres Strait Islander Australians (hereafter respectively termed ‘Indigenous’) comprise 3.3% of the total Australian population [1]. They represent a rich and diverse culture, one which is steeped in an abundance of customs and languages. Prior to European settlement in the 1780s, the health and wellbeing of Indigenous Australians was characterised by physical strength and agility, diets free of refined carbohydrates, and environmental practises which had been sustained for approximately 65,000 years [2]. The rapid changes in lifestyle and diet, and sustained government policies of assimilation, discrimination and land dispossession, has had grave consequences on the health and wellbeing of contemporary Indigenous Australians [3].

Oral health is an integral part of general health. Dental caries and periodontal disease can cause debilitating pain, and result in tooth loss. This, in turn, may lead to difficulties with eating and speaking, embarrassment regarding aesthetics, inadequate masticatory capacity, and reduced nutrient intake due to changes in diet [4–6]. Dental diseases are associated with many chronic diseases, including type 2 diabetes [7], cardiovascular disease [8], kidney disease [9], and Alzheimer’s disease [10]. Global self-ratings of oral health provide a useful insight into how patients view their oral health in a holistic sense, one which is unique and distinct from clinical diagnoses [11].

Health-related quality of life is the perceived quality of an individual’s well-being or lack thereof. It includes emotional, social, and physical aspects of an individual’s health. Instruments assessing health-related quality of life are commonly used as proxy measures of general health, and are represented by quality weights (utilities) [12]. Over 1000 instruments/ tools are available to assess quality of life [13], with the most commonly used being the.

‘Short-Form 12-Item Health Survey version 2 (SF-12v2)’ instrument [14] to measure Health-Related Quality of Life (HRQOL), the World Health Organization Quality of Life Instruments (WHOQOL-BREF) [15], and EuroQol (EQ-5D or EQ-5D-5L) [12, 16]. EQ-5D, a measure used to calculate quality adjusted life years (QALYs) to guide economic evaluations, is composed of five domains. Each dimension in the EQ-5D has been subdivided into five levels (5 L) to form EQ-5D-5L. EQ-5D is a widely utilised multi-attribute utility instrument used for estimating utility, and has been used to assess health-related quality of life among Indigenous populations, both in Australia [17, 18] and elsewhere [19, 20]. However, to the best of our knowledge EQ-5D-5L has not been used among the Indigenous Australian population to examine oral health outcomes.

Working in partnership with seven Indigenous communities in South Australia, this study aimed to: 1) quantify self-rated oral health and health-related quality of life and; 2) investigate associations between poor self-rated oral health and general health among Indigenous Australian adults.

## Methods

Data was obtained from a large convenience sample (*n* = 1011) of Indigenous Australian adults in South Australia aged 18+ years between Feb 2018 and Jan 2019, as part of a broader study [21]. This study had the oversight of an Indigenous Reference Group (IRG), who advised on all aspects of project staff employment, participant recruitment, data collection, analysis and feedback. In the absence of a specific instrument to capture health-related quality of life through an Indigenous lens, the IRG endorsed items in the EQ-5D-5L as being an acceptable representation of their views of health and wellbeing. This was then pilot tested among eight Indigenous participants, who provided similar feedback. Validity and reliability of the EQ-5D-5L in the Indigenous Australian context has recently been assessed (in press). Participants were recruited through Aboriginal Community Controlled Health Organisations (ACCHOs), who were additional key stakeholders in the study.

### Data collection

Data collection included face-to-face interviews by experienced research officers who were trained and calibrated in their delivery of the study aims, and managed by a senior Indigenous research officer. The self-report questionnaire included items pertaining to socio-demographic characteristics, self-rated oral health, oral-health related behaviours and health-related quality of life.

### Variables

The outcome variable was general health-related quality of life (general health), which was assessed using the EuroQoL instrument (EQ-5D-5L) [12]. The EQ-5D-5L is one of the most popular, validated tools for estimating general health-related quality of life. There are 5 dimensions (5D): mobility, self-care, usual activities, pain/discomfort and anxiety/ depression. Each dimension of the EQ-5D has five levels (5 L), with increasing level numbers corresponding to increasing levels of problems, described as: no problems; slight; moderate; severe; and extreme problems. A total possible 3125 health states were measured by combining one level from each dimension, ranging from the worst state (55555), five mild states (21,111, 12,111, 11,211, 11,121, and 11,112) to the full health state (11111), and evaluated by converting into a single index utility score using a scoring algorithm based on public preferences. To date, there are no values specific to the (five-level) EQ-5D-5L in Australia to summarise population data (and certainly none for the Indigenous Australian population), therefore the UK algorithm was used to calculate utility scores [12]. A small study in New Zealand (*n* = 66) tested validity of the EQ-5D-3L among Maori [20]. The conclusions were that, while test-retest reliability was demonstrated, evidence of construct validity was not possible due to high numbers of missing values. An individual’s overall utility score can be obtained by the full health score subtracting the score corresponding to each dimension’s response level. For instance, the utility value ‘53124’ as a health state means ‘extreme problems with mobility, moderate problems with self-care, no problems with usual activities, slight problems with pain/discomfort and severe problems with anxiety/depression’, should be 0.298, i.e. 1.000 (full health) minus 0.274 (mobility level 5) minus 0.080 (self-care level 3) minus 0 (usual activities level 1) minus 0.063 (pain/ discomfort level 2) minus 0.285 (anxiety/ depression level 4) [12]. This study was focused on the poor general health status, so the disutility scores were used and calculated by 1 minus utility score correspondingly [12, 16]. Higher disutility scores indicate worsening general health. In addition, pooled responses from the full health status (11111) to the worst state (55555) of the five dimensions of EQ-5D and were classified as ‘no problems’ or ‘at least one problem’ to explore association between individual EuroQoL dimensions and exposure variables.

The main exposure variable was self-rated oral health, which was assessed by the item ‘Would you rate your oral health as…’ with the five response levels dichotomised into ‘fair/poor’ and ‘excellent/very good /good’. This is a global rating of oral health that has been used in population oral health surveys in Australia, the United Kingdom, New Zealand and many other Organisation for Economic Co-operation and Development (OECD) countries. Covariates included socio-demographic characteristics and health behaviours. The socio-demographic characteristics were ‘Age’; dichotomised two groups (> 50 vs ≤ 50 years); ‘Sex’ (Male vs Female); ‘Geographic location’ (‘Metropolitan’ vs ‘Non-metropolitan’); ‘Highest educational attainment’ (dichotomised into ‘High school or less’ vs ‘Trade/TAFE/University’; TAFE stands for ‘Technical and Further Education’ and provides training for vocational occupations); ‘Income’ (defined as ‘Job’ vs ‘Welfare support payments’); ownership of a Government-administered health care card (HCC) (Yes vs No; a HCC is means-tested, and enables access to services such as publicly-funded dental care). The health-related behaviours included ‘Tobacco smoking status’ and ‘Non-prescription tobacco substitute (vape, e-cigarette)’. These were defined as ‘current smoker’, ‘ex-smoker’ and ‘never smoked’. ‘Recreational drug use’ was classified as ‘Currently use’, ‘Don’t now but used to’ and ‘Never used’.

### Data analysis

Descriptive analyses were conducted to examine the distributions of socio-demographic, oral health and health-related behaviours, as well as (dis)utility scores. This was followed by bivariate analyses to describe associations with poor general health by estimating severity (mean disutility score) and prevalence (% having at least one problem for individual dimensions) of EQ-5D-5L with their 95% confidence intervals (95% CI). Differences were denoted to be statistically significant when 95% CI did not overlap. Multivariable log-Poisson regression models were used to estimate associations between poor self-rated oral health and poor general health by calculating mean rate ratios (MRR) for disutility scores, and prevalence ratios (PR) for individual dimensions, after adjusting for social-demographic characteristics and health-related behaviours.

## Results

Data were available for 1011 Indigenous South Australians aged 18+ years who completed the self-rated oral health and EQ-5D-5L items. The mean age was 39.8 years (95% CI: 38.9–40.7) and the mean utility score was 0.82 (95% CI: 0.81–0.83).

The sample socio-demographic characteristics and mean disutility scores are summarised in Table 1. A higher proportion of the study sample were in the younger age group (≤ 50 years), were female, owned a health care card, resided in non-metropolitan locations, received ‘high school or less’ education, relied on welfare for income, reported being current smokers of tobacco, never using non-prescription tobacco and recreational drugs, and rated their oral health as ‘excellent/very good or good’.

**Table 1:** Sample characteristics and disutility score (EQ-5D-5L) among Indigenous Australian adults

	Number	Percentage	Disutility score
		% (95 CI)	Mean (95% CI)
**Total**	1011	100	0.18 (0.17–0.19)
**Self-rated Oral health**			
Fair/Poor	329	33.5 (30.5–36.4)	**0.25 (0.22–0.27)**
Excel/Very good/Good	654	66.5 (63.6–69.5)	**0.15 (0.13–0.16)**
**Age groups (Years)**			
> 50	283	28.0 (25.2–30.8)	**0.26 (0.22–0.29)**
≤ 50	728	72.0 (69.2–74.8)	**0.15 (0.14–0.17)**
**Sex**			
Male	340	33.6 (30.7–36.5)	0.17 (0.15–0.19)
Female	671	66.4 (63.5–69.3)	0.19 (0.17–0.20)
**Geographic location**			
Non-metropolitan	633	62.7 (59.7–65.7)	0.17 (0.15–0.19)
Metropolitan	376	37.3 (34.3–40.3)	0.20 (0.18–0.22)
**Level of Education**			
High school or less	679	68.2 (65.3–71.1)	0.17 (0.16–0.19)
Trade/TAFE/University	317	31.8 (28.9–34.7)	0.20 (0.18–0.22)
**Income**			
Welfare support payments	757	76.0 (73.3–78.7)	**0.19 (0.18–0.21)**
Job	239	24.0 (21.3–26.7)	**0.14 (0.12–0.17)**
**Health Care Card ownership**			
Yes	762	79.0 (76.4–81.5)	**0.19 (0.18–0.21)**
No	203	21.0 (18.5–23.6)	**0.15 (0.12–0.17)**
**Smoke status**			
Current smoker	568	59.4 (56.3–62.5)	0.19 (0.17–0.21)
Ex-smoker	113	11.8 (9.8–113.9)	0.21 (0.17–0.26)
Never smoked	275	28.8 (25.9–31.6)	0.16 (0.13–0.18)
**Use of non-prescription tobacco substitutes (vape, e-cigarette)**			
Currently smoke	116	12.1 (10.0–14.2)	0.19 (0.15–0.22)
Don’t now but used to	182	19.0 (16.5–21.5)	0.17 (0.15–0.20)
Never smoked	660	68.9 (66.0–71.8)	0.18 (0.16–0.19)
**Use of recreational drugs**			
Currently use	208	20.9 (18.3–23.4)	**0.21 (0.18–0.23)**
Don’t now but used to	333	33.4 (30.5–36.3)	0.20 (0.18–0.22)
Never used	456	45.7 (42.6–48.8)	**0.16 (0.14–0.18)**

The mean utility score of the sample (0.82) corresponds to a total mean disutility score of 0.18 (95% CI: 0.17–0.19). Higher disutility scores were observed among those rated their oral health as ‘fair or poor’ (0.25, 95% CI: 0.22–0.27), in the older age group (0.26, 95% CI: 0.26–0.29), who relied on welfare for income (0.19, 95%CI: 0.18–0.21), owned a health care card (0.19, 95%CI: 0.18–0.21), were current drug users (0.21, 95% CI: 0.18–0.23), comparing with their counterparts. (Table 1).

Overall, the prevalence of at least some problems for the five dimensions of EQ-5D ranged from less than 10% (self-care) to almost 60% (anxiety/ depression) (Table 2). Indigenous Australians with fair/poor self-rated oral health had 1.5 to 2.5 times higher prevalence of problems (Level > 1 for at least one domain) of the five EQ-5D dimensions than those rating their oral health as ‘excellent, very good or good’. Consistent with the sample overall, the highest prevalence (71.3%) of at least one problem was reported for the anxiety/ depression dimension.

**Table 2:** Bivariate association with prevalence of at least one problem for five dimensions of EQ-5D (*n* = 1011)

	Mobility: at least one problem	Self-care: at least one problem	Usual activities: at least one problem	Pain/discomfort: at least one problem	Anxiety/depression: at least one problem
	% (95% CI)	% (95% CI)	% (95% CI)	% (95% CI)	% (95% CI)
**Total**	28.6 (25.8–31.4)	9.0 (7.2–10.8)	24.8 (22.1–27.5)	52.2 (49.1–55.3)	58.3 (55.2–61.3)
**Self-rated oral health**					
Fair/Poor	**42.9 (37.6–48.3)**	**14.7 (10.8–18.5)**	**35.0 (29.8–40.1)**	**66.8 (61.6–71.9)**	**71.3 (66.4–76.2)**
Excel/Very good/Good	**21.2 (18.1–24.3)**	**6.0 (4.2–7.8)**	**19.4 (16.3–22.4)**	**45.1 (41.2–48.9)**	**51.8 (48.0–55.7)**
**Age groups (Years)**					
> 50	**50.5 (44.7–56.4)**	**16.8 (12.4–21.2)**	**40.8 (35.0–46.5)**	**65.1 (59.5–70.7)**	58.4 (52.6–64.2)
≤ 50	**20.0 (17.1–22.9)**	**6.0 (4.2–7.7)**	**18.6 (15.7–21.4)**	**47.1 (43.5–50.8)**	58.2 (54.6–61.8)
**Sex**					
Male	27.3 (22.5–32.1)	9.2 (6.1–12.3)	24.9 (20.2–29.5)	51.3 (46.0–56.7)	55.7 (50.3–61.0)
Female	29.2 (25.8–32.7)	8.9 (6.7–11.1)	24.8 (21.5–28.1)	51.6 (48.8–56.4)	59.6 (55.8–63.3)
**Geographic location**					
Non-metropolitan	28.3 (24.8–31.9)	8.7 (6.4–10.9)	23.8 (20.5–27.1)	**48.5 (44.5–52.4)**	**52.8 (48.9–56.7)**
Metropolitan	29.1 (24.5–33.8)	9.6 (6.6–12.6)	26.7 (22.2–31.2)	**58.3 (53.3–63.4)**	**67.5 (62.7–72.2)**
**Level of Education**					
High school or less	27.4 (24.1–30.8)	9.2 (7.0–11.4)	23.0 (19.9–26.2)	**48.3 (44.5–52.1)**	55.9 (52.2–59.7)
Trade/TAFE/University	31.0 (25.9–36.1)	8.6 (5.5–11.7)	28.9 (23.9–33.9)	**61.0 (55.6–66.3)**	64.1 (58.8–69.4)
**Income**					
Welfare support payments	**31.0 (27.7–34.3)**	**10.2 (8.1–12.4)**	**27.6 (24.4–30.8)**	52.2 (48.6–55.8)	59.2 (56.0–63.4)
Job	**19.7 (14.6–24.7)**	**5.0 (2.2–7.8)**	**16.3 (11.6–21.0)**	51.9 (45.5–58.2)	56.5 (50.2–62.8)
**Health Care Card ownership**					
Yes	**31.2 (27.9–34.5)**	**10.7 (8.5–12.9)**	**27.0 (23.8–30.2)**	50.8 (47.2–54.4)	57.8 (54.3–61.3)
No	**18.2 (12.9–23.5)**	**3.0 (0.6–5.3)**	**18.2 (12.9–23.5)**	58.4 (51.6–65.2)	61.4 (54.7–68.1)
**Smoke status**					
Current smoker	28.3 (24.6–32.0)	9.6 (7.2–12.0)	25.7 (22.1–29.3)	52.7 (48.5–56.8)	**61.6 (57.5–65.6)**
Ex-smoker	37.2 (28.2–46.1)	8.8 (3.6–14.1)	27.4 (19.2–35.7)	61.1 (52.1–70.1)	61.9 (53.0–70.9)
Never smoked	25.5 (20.3–30.6)	8.0 (4.8–11.3)	22.2 (17.3–27.1)	48.9 (43.0–54.8)	**50.9 (45.0–56.9)**
**Use of non-prescription tobacco substitutes (vape, e-cigarette)**					
Currently smoke	26.5 (18.4–34.7)	10.6 (4.9–16.3)	28.1 (19.8–36.3)	49.6 (40.4–58.7)	64.2 (55.6–73.1)
Don’t now but used to	26.9 (20.5–33.4)	9.1 (5.1–13.6)	26.9 (20.5–33.4)	52.5 (45.2–59.8)	61.9 (54.8–69.0)
Never smoked	29.2 (25.7–32.7)	7.9 (5.8–10.0)	23.7 (20.4–26.9)	52.0 (48.2–55.8)	56.2 (52.4–60.0)
**Use of recreational drugs**					
Currently use	27.8 (21.7–33.9)	9.2 (5.3–13.2)	28.0 (21.9–34.1)	56.0 (49.3–62.8)	**70.4 (64.1–76.6)**
Don’t now but used to	28.6 (23.7–33.5)	10.8 (7.5–14.2)	27.7 (22.9–32.5)	56.0 (50.6–61.4)	**66.1 (60.9–71.2)**
Never used	29.1 (24.9–33.3)	7.5 (5.1–9.9)	21.4 (17.6–25.1)	48.0 (43.4–52.6)	**47.7 (43.1–52.3)**

Prevalence of at least one problem was higher in the older age group (> 50 years) than in the younger age group (≤ 50 years) for all dimensions except anxiety/ depression, and the highest prevalence was 65% for pain/discomfort. The prevalence of at least one problem was also higher among those dependent on welfare for income, and those with an HCC (with mobility around 31%, self-care around 10% and usual activities around 27% in both cases), comparing with their counterparts. The prevalence of at least one problem was higher among current smokers and recreational drug users. A higher prevalence of at least one problem was also reported among metropolitan-dwelling participants (for pain/discomfort (58%) and anxiety/depression (68%)), and ‘Trade/TAFE/University’ education (for pain/discomfort; 61%)).

Fair/poor self-rated oral health was positively associated with overall total disutility score, as well as each dimension of EQ-5D (Table 3). After adjusting for all covariates, the mean disutility score was 1.6 times higher, and the prevalence of at least one problem ranged from 87 to 162% higher risk across each individual EQ-5D dimension among those reporting fair/poor self-rated oral health. Older age and current drug use were also significantly associated with total disutility and most individual EQ-5D dimensions. Factors significantly associated with one or more problems in the EQ-5D dimensions included: self-care with health care card ownership and usual activities with income; pain/discomfort with metropolitan residential location; and anxiety/depression with male, sex and metropolitan residential location.

**Table 3:** Multivariable regression modelling for total disutility scores (Mean rate ratio: MRR) and at least one problem for 5 dimensions (prevalence ratio: PR) of EQ-5D (*n* = 1011)

	Disutility scores	Mobility	Self-care	Usual activities	Pain/Discomfort	Anxiety/Depression
	MRR (95% CI)	PR (95% CI)	PR (95% CI)	PR (95% CI)	PR (95% CI)	PR (95% CI)
**Self-rated oral health**						
Fair/Poor	**1.56 (1.12–2.17)**	**2.53 (1.81–3.56)**	**2.62 (1.55–4.41)**	**1.87 (1.32–2.64)**	**2.24 (1.63–3.07)**	**2.41 (1.73–3.36)**
Excel/Very good/Good	ref	ref	ref	ref	ref	ref
**Age groups (Years)**						
> 50	**1.60 (1.12–2.31)**	**3.88 (2.26–5.62)**	**2.75 (1.57–4.80)**	**2.79 (1.91–4.07)**	**2.05 (1.44–2.93)**	1.15 (0.81–1.65)
≤ 50	ref	ref	ref	ref	ref	ref
**Sex**						
Male	0.86 (0.60–1.25)	0.93 (0.64–1.36)	1.07 (0.61–1.89)	0.94 (0.64–1.36)	0.86 (0.62–1.19)	**0.63 (0.45–0.87)**
Female	ref	ref	ref	ref	ref	ref
**Geographic location**						
Non-metropolitan	0.85 (0.61–1.19)	0.93 (0.66–1.31)	0.87 (0.51–1.48)	0.88 (0.62–1.23)	**0.68 (0.51–0.93)**	**0.57 (0.42–0.79)**
Metropolitan	ref	ref	ref	ref	ref	ref
**Level of Education**						
High school or less	0.93 (0.65–1.34)	0.73 (0.51–1.06)	1.01 (0.57–1.80)	0.71 (0.49–1.03)	0.72 (0.52–1.00)	0.93 (0.66–1.31)
Trade/TAFE/University	ref	ref	ref	ref	ref	ref
**Income**						
Welfare support payments	1.15 (0.68–1.96)	1.33 (0.78–2.27)	1.14 (0.47–2.77)	**1.83 (1.05–3.17)**	1.18 (0.76–1.83)	0.98 (0.62–1.54)
Job	ref	ref	ref	ref	ref	ref
**Health Care Card ownership**						
Yes	1.11 (0.65–1.90)	1.62 (0.93–2.81)	**5.67 (1.53–21.0)**	1.06 (0.61–1.82)	0.68 (0.44–1.07)	0.86 (0.55–1.36)
No	ref	ref	ref	ref	ref	ref
**Smoke status**						
Current smoker	1.04 (0.69–1.58)	1.21 (0.80–1.85)	0.97 (0.51–1.83)	1.05 (0.69–1.61)	1.00 (0.70–1.43)	1.07 (0.74–1.54)
Ex-smoker	1.07 (0.61–1.87)	1.42 (0.80–2.50)	0.59 (0.21–1.61)	0.76 (0.42–1.39)	1.16 (0.69–1.94)	0.99 (0.58–1.67)
Never smoked	ref	ref	ref	ref	ref	ref
**Use of non-prescription tobacco substitutes (vape, e-cigarette)**						
Currently smoke	0.94 (0.56–1.60)	0.83 (0.48–1.44)	1.19 (0.54–2.65)	1.02 (0.59–1.76)	0.77 (0.48–1.24)	0.97 (0.59–1.60)
Don’t now but used to	0.87 (0.56–1.36)	0.85 (0.54–1.33)	0.89 (0.44–1.80)	1.21 (0.77–1.88)	0.93 (0.63–1.37)	1.04 (0.69–1.55)
Never smoked	ref	ref	ref	ref	ref	ref
**Use of recreational drugs**						
Currently use	1.33 (0.83–2.13)	0.87 (0.53–1.43)	0.95 (0.42–2.13)	1.52 (0.93–2.49)	**1.73 (1.14–2.64)**	**2.75 (1.78–4.28)**
Don’t now but used to	**1.61 (1.06–2.43)**	**1.57 (1.04–2.38)**	**2.51 (1.31–4.81)**	**2.34 (1.52–3.60)**	**2.11 (1.46–3.06)**	**2.67 (1.84–3.89)**
Never used	ref	ref	ref	ref	ref	ref

## Discussion

This study is the first to evaluate general health-related quality of life - using EQ-5D 5 L, and to estimate associations with self-rated oral health, among Indigenous Australians. Our findings demonstrated that poorer self-rated oral health was associated with higher disutility scores, as well as higher prevalence of at least one problem in each of the five EQ-5D dimensions (indicating poorer general health). Being older, male, residing in metropolitan locations, relying on the government for income, ownership of a health care card, never consuming alcohol and current recreational drug use were also significantly associated with high disutility scores and/or individual EQ-5D dimensions, after adjustment in multivariable models.

It is important to acknowledge that, overall, the degree of utility was low (0.82) in the study compared with estimates from the non-Indigenous Australian population (0.91) [16]. This suggests the general health-related quality of life among Indigenous Australians in our study is, on the whole, poorer than the general Australian population. This finding is in keeping with many studies and national health reports [22, 23] suggesting that Indigenous Australians score worse on almost every indicator of health well-being relative to non-Indigenous populations. Our findings provide evidence of poorer general health (based on EQ-5D-5L) for the Indigenous Australian population compared with the Australian population overall.

Our findings also indicate that poor self-rated oral health is associated with poor general health-related quality of life among Indigenous Australians. The findings are consistent with previous studies for the general population [6, 24, 25]. In a representative population in South Australia, people with less than 21 teeth had 2 times higher disutility, and had 26 and 16% higher risk of reporting at least one problem in mobility and pain/discomfort respectively, after adjusting for other covariates [24]. Other evidence suggests that health-related quality of life improves for patients who receive root canal treatment, compared with those had tooth extraction [6]. Xerostomia (dry mouth, usually caused by medication) is usually associated with poor oral and general health [25]. This has been attributed to many factors, including the social determinants of health, long-term impacts of colonisation, dental/health service provision models that do not favour Indigenous holistic models of care, and inequitable access to dental/health services in part the consequence of regional and remote geographic location of residence (refs), results in increasing psychological distress, trauma, socioeconomic disadvantage, and high burden of chronic diseases.

Although EQ-5D has been used to assess health-related quality of life among Indigenous Australians, the need for health-related quality of life instruments that are specifically tailored for Indigenous populations is recognised [26, 27]. Indigenous conceptions of health differ from the conceptions of non-Indigenous persons in fundamental ways that are not captured by generic quality of life instruments. Constructs that lie outside the five EuroQoL domains are not taken into account, such as social, cultural, diet, land use, community and experiences of dispossession, colonisation, racism and assimilation domains, We acknowledge that the EuroQoL instrument captures only a small component of what constitutes health in the Indigenous lens, but chose to use it in the absence of another instrument that had been tested and validated in Australia at the time of the study, and after endorsement by the study’s IRG. It is interesting to note that Perkins and colleagues [20] reported that over three quarters of their Maori sample in New Zealand agreed that the EQ-5D adequately represented their concept of health. Angell and colleagues [26] also noted that some existing HRQoL measures were used in Indigenous populations, with four instruments deemed to be valid (excluding EQ-5D or EQ-5D-5L). Moving forward, it is essential that the value of health-related quality of life Indigenous populations is encapsulated in a manner that better reflects all of those aspects of health which are important for Indigenous people.

Strengths of this study include: 1) it is the first to work in partnership with the Indigenous Australian community to report self-rated oral health and health-related quality of life; 2) a large representation of the broader South Australian Indigenous population (*n* = 1011, Table 1) is critical, with the findings potentially having meaning for other Indigenous groups residing in similar socio-economic regions of the world. Limitations of this study include the lack of oral clinical examination data, with no ability to correlate actual clinical dental disease status against impacts on daily life. In addition, our findings could be affected due to the proportion of older age (> 50 years), female, non-metropolitan location, welfare-based income, health care card holder and currently smoking being higher in this South Australian Indigenous population than in broader Indigenous population (see Supplements Table S1).

In conclusion, our findings indicate that general health-related quality of life was lower among the sampled Indigenous population than previously reported for non-Indigenous populations, and also that poor self-rated oral health was associated with poor general health in this Indigenous population, including across all five dimensions assessed in EQ-5D. Most oral health conditions are preventable and can be treated in their early stages. Our findings therefore suggest that improving oral health would also improve broader aspects of health-related quality of life. Our findings are an important contribution to cost-utility (such as measurements of utilities for more specific oral health states: dental caries, periodontal disease and oral cancers) and disease prevention strategies that seek to inform policies around improving oral health among all Australians.

## Supplementary Information

**Additional file 1: Table S1.** Socio-demographic characteristics, health-related behaviours including tobacco, alcohol and drug use (percent, 95% CI) and compared against population estimates in 2016.

## Data Availability

The datasets generated and/or analyzed during the current study are not publicly available due to privacy issues of the participants. Data are available from the corresponding author on reasonable request.

## Abbreviations

ACCHOs: Aboriginal Community Controlled Health Organisations
CI: Confidence intervals
EuroQoL: Europe Quality of life
EQ-5D-5L: EuroQoL-five dimensions-five levels
HCC: Health care card
HRQOL: Health-Related Quality of Life
IRG: Indigenous Reference Group
MRR: Mean rate ratio
OHRQoL: Oral health-related quality of life
PR: Prevalence ratio
QALYs: Quality adjusted Life Years
TAFE: Technical and Further Education
WHOQO-BREF: World Health Organization Quality of Life
