# Supplementary material for: Poor self-rated oral health associated with poorer general health among Indigenous Australians
Source: BMC Public Health. 2021 Mar 1;21:424. doi: 10.1186/s12889-021-10426-3 (PMC7919297; doi:10.1186/s12889-021-10426-3)
Supplement: Supplementary file 1 — Additional file 1: Table S1. Socio-demographic characteristics, health-related behaviours including tobacco, alcohol and drug use (percent, 95% CI) and compared against population estimates in 2016. [file 12889_2021_10426_MOESM1_ESM.docx]

Table S1: Socio-demographic characteristics, health-related behaviours including tobacco, alcohol and drug use (percent, 95% CI) and compared against population estimates in 2016

|  | HPV-OPC study ^c^ | 2016 South Australian Census/other surveys |
| --- | --- | --- |
| **Age groups (Years)** |  |  |
| >50 | 28.0 (25.2-30.8) | ^a^24.5 (24.4-24.6) |
| ≤ 50 | 72.0 (69.2-74.8) | 75.5 (75.4-75.6) |
| **Sex** |  |  |
| Male | 33.6 (30.7-36.5) | ^a^48.4 (47.7-49.1) |
| Female | 66.4 (63.5-69.3) | 51.6 (50.9-52.3) |
| **Geographic location** |  |  |
| Non-metropolitan | 62.7 (59.7- 65.7) | ^a^38.0 (37.4-38.7) |
| Metropolitan | 37.3 (34.3 -40.3) | 62.0 (61.3-62.6) |
| **Level of Education** |  |  |
| High school or less | 68.2 (65.3-71.1) | ^a^ 65.5 (64.8-66.3) |
| Trade/TAFE/University | 31.8 (28.9-34.7) | 34.5 (33.8-35.2) |
| **Income** |  |  |
| Welfare support payments | 76.0 (73.3-78.7) | ^a^65.5 (64.8-66.3) |
| Job | 24.0 (21.3-26.7) | 34.5 (33.8-35.2) |
| **Health Care Card ownership** | |  |
| Yes | 79.0 (76.4-81.5) | ^a^57.0 (56.3-57.7) |
| No | 21.0 (18.5-23.6) | 43.0 (42.3-43.7) |
| **Smoke status** |  |  |
| Current smoker | 59.4 (56.3-62.5) | ^b^43.3 (39.2-47.4) |
| Ex-smoker | 11.8 (9.8-11.9) | 24.6 (21.1-28.2) |
| Never smoked | 28.8 (25.9-31.6) | 32.7 (28.9-36.6) |
| **Use of recreational drugs** |  |  |
| Currently use | 20.9 (18.3-23.4) | ^b^26.9 (23.2-31.1) |
| Don’t now but used to | 33.4 (30.5-36.3) | 27.6 (24.0-31.3) |
| Never used | 45.7 (42.6-48.8) | 45.2 (41.1-49.3) |

**Notes:**

1. 2016 Census for South Australian Indigenous population
2. Australian Institute of Health and Welfare 2017. National Drug Strategy Household Survey 2016: detailed findings. Drug Statistics series no. 31. Cat. no. PHE 214. Canberra: AIHW. (for Australian Indigenous population)
3. Jamieson L, Garvey G, Hedges J, Mitchell A, Dunbar T, Leane C, et al. Human Papillomavirus and Oropharyngeal Cancer Among Indigenous Australians: Protocol for a Prevalence Study of Oral-Related Human Papillomavirus and Cost-Effectiveness of Prevention. JMIR Res Protoc. 2018;7(6): e10503.
